# A novel survey of raptor collections in Europe and their potential to provide samples for pan-European contaminant monitoring

**DOI:** 10.1007/s11356-021-16984-8

**Published:** 2021-10-16

**Authors:** Gloria Ramello, Guy Duke, Rene W. R. J. Dekker, Steven van der Mije, Paola Movalli

**Affiliations:** 1 Museo Civico Di Storia Naturale, Cascina Vigna, via San Francesco di Sales 188, 10022 Carmagnola, TO Italy; 2 Environmental Change Institute, Oxford, OX1 3QY UK; 3 Naturalis Biodiversity Center, Darwinweg 2, 2333 CR Leiden, the Netherlands

**Keywords:** Raptor, Collection, Natural history museum, Environmental specimen bank, Contaminant, Monitoring

## Abstract

This paper provides a novel survey of current collections of frozen raptor carcasses and tissue samples in natural history museums (NHMs), environmental specimen banks (ESBs) and other research collections (ORCs e.g. at universities and research institutes) across Europe and assesses the extent to which collections might support pan-European raptor biomonitoring through the provision of samples for contaminant analyses. The paper is based on questionnaire responses received in late 2018 and early 2019 from 116 institutions. Issues covered include the number of raptor carcasses and diversity of raptor species arriving annually at collections, the number of carcasses stored in freezers, the extent to which collections retain frozen tissue samples, what records are kept of carcasses and tissue samples, constraints to expanding collections of frozen carcasses and tissues and the extent to which collections currently engage in ecotoxicological research and monitoring. Our findings show that collections in Europe receive well over 5000 raptor carcasses per annum, and that NHMs are the key recipients of raptor carcasses for most countries. Collections in Europe probably hold well over 10,000 raptor carcasses in their freezers, offering a substantial resource of frozen raptor carcasses and tissues from recent years. Moreover, these carcasses include good specimen numbers for species that have been prioritized for pan-European contaminant monitoring. Collections are becoming digitized aiding access to samples. However, freezer capacity is a key constraint to retention of carcasses, and contaminant biomonitoring is novel for most NHMs. Our findings on the repository and availability of frozen raptor carcasses and tissues held by collections in Europe can enable greater use of these specimens for pan-European contaminant monitoring in support of better chemicals management. We highlight opportunities to further optimize raptor collections for pan-European contaminant monitoring.

## Introduction

This paper presents a novel survey of current collections of frozen raptor carcasses and tissue samples in natural history museums (NHMs), environmental specimen banks (ESBs) and other research collections (ORCs e.g. universities and research institutes) across Europe. This survey was carried out through the COST Action ‘European Raptor Biomonitoring Facility’. COST Actions are research networks funded by the European Cooperation in Science and Technology (COST). The survey covers the geographical area of the COST Member Countries — which includes all countries of the European continent except Russia, Ukraine, Belarus and Moldova — plus Israel (a COST Cooperating Member).

The objective of the survey was to understand to what extent NHMs, ESBs and ORCs (together referred to hereinafter as ‘collections’) might support contemporary pan-European raptor biomonitoring through the provision of frozen tissues for contaminant analyses. Issues covered by the survey include the number of raptor carcasses and diversity of raptor species arriving annually at collections, the number of carcasses stored in freezers, the extent to which collections retain frozen tissue samples, what records are kept of these carcasses and tissue samples, constraints to expanding such collections of frozen carcasses and tissues and the extent to which collections currently engage in ecotoxicological research and monitoring.

The focus of this survey is on raptor carcasses and tissue samples stored in freezers, which can be of particular value for monitoring of present-day environmental contaminants (Espín et al. 2016), notably chemicals of emerging concern (e.g. pharmaceuticals, personal care products, flame retardants) but also legacy contaminants (e.g. persistent organic pollutants such as dichlorodiphenyltrichloroethane [DDT] and dieldrin). Better knowledge of the repository and availability of frozen raptor carcasses and tissues scattered across collections in Europe can enable the use of these specimens for pan-European contaminant monitoring.

We do not address in this survey NHMs’ archive collections of dry tissues (skins, bones), as these are less pertinent to monitoring of present-day contaminants, although they can be of great value to derive contaminant reference values for legacy contaminants.

For the purposes of this study, raptors (birds of prey) include both diurnal — Accipitridae (hawks, eagles, buzzards, harriers, kites and Old World vultures), Pandionidae (osprey) and Falconidae (falcons) — and nocturnal species — Strigidae (typical owls) and Tytonidae (barn owl). They are particularly well suited to contaminant monitoring (Movalli et al. 2008, 2017, 2018; Vrezec et al. 2012).

Monitoring of contaminants in raptors can provide early warning of emerging contaminant problems in the environment, inform risk assessment of chemicals and inform evaluation of the effectiveness of chemicals risk management measures (Movalli et al. 2017). These applications can enable improvements in the management of chemicals in Europe and thereby help to deliver improved environmental and human health, which is the ultimate objective of EU chemicals regulations (e.g. European Commission 2001a, b, 2004a, b, 2006, 2009, European Union 2012).

This survey is an important first step towards the development of a distributed European Raptor Specimen Bank for the reliable provision of frozen raptor samples for coordinated pan-European contaminant monitoring. It supports ongoing work: (1) to bring collections of frozen raptor carcasses and tissue samples together through a pan-European database, thereby making them more visible and accessible to ecotoxicologists and competent authorities (Movalli et al. 2019); (2) to enhance quality standards for the gathering, processing and storage of raptor carcasses and tissues in collections and (3) to support the engagement of collections in contaminant monitoring using raptors. The pan-European database is being taken forward in dialogue with the Distributed System of Scientific Collections (DiSSCo) to ensure interoperability, and will be linked also to data on individual raptor tissue samples and contaminant data arising from non-target screening, wide-scope target screening and targeted analyses of these samples, housed by the NORMAN database.^1^ Contaminant data in the NORMAN database is in turn linked to IPCHEM, the European Commission’s portal for data on environmental contaminants.^2^

Development of a distributed European Raptor Specimen Bank is one of three key elements making up the European Raptor Biomonitoring Facility (ERBFacility).^3^ The other two elements are the following: (1) a European Raptor Biomonitoring Scheme, which will specify priority species and matrices for contaminant monitoring and (2) a European Raptor Sampling Programme, which will provide the framework for ongoing sourcing of raptor specimens from the field. ERBFacility will thus ensure a strategic approach to the supply of raptor specimens from the field, their processing and storage in collections and subsequent contaminant analysis of samples, thus providing the data needed by regulators to inform better chemicals management in Europe. Considerable other work has been carried out towards pan-European contaminant monitoring in raptors (e.g. Gómez-Ramírez et al. 2014; Espín et al. 2016, 2020; Badry et al. 2020; Monclús et al. 2020; González-Rubio et al. 2021).

## Materials and method

We conducted the survey in five steps:

1. Gathering contact details for relevant collections.
2. Developing and formatting of a *Google Forms* questionnaire.
3. Issuing the questionnaire and supporting documents.
4. Tracking and encouraging responses.
5. Analysis of response data, development of discussion and conclusions.

The survey of raptor collections was carried out through the development and circulation of an online questionnaire.

### 1: Gathering contact details for relevant collections

We first prepared a contact list for relevant collections in Europe. We identified NHMs with raptor collections using the *Electronic Inventory of European Bird Collections* on the electronic bulletin board for European avian curators (eBEAC).^4^ We downloaded eBEAC’s list of contacts and added to this relevant ESBs and ORCs known to house raptor carcasses/tissues, based on the authors’ personal networks or identified through web search. We checked contact details for each collection by web search (Google), using keywords such as ‘contact’, ‘staff”, ‘curator’ and ‘curator of bird collections’.

### 2. Development and formatting of a Google Forms questionnaire

We developed a questionnaire structured in 5 parts:

A. Contact information.
B. Receipt/collection of fresh (contemporary) specimens and storage of samples from these specimens. Questions under this heading addressed, among other issues, whether the collection receives and stores fresh raptor specimens and, if so, from whom, how many, from what regions(s), whether specimens are frozen for storage, whether records are kept of specimens stored, how many carcasses are currently stored in freezers and of which species, whether skins and/or other tissues from carcasses are added to the archive and, if so, which tissues and how these are stored and whether, if freezer space is limited, carcasses are discarded and/or made available to other institutions.
C. Constraints to receiving/collecting, processing and storage of fresh specimens.
D. Historical archives.
E. Related studies.

Questions were structured as multiple-choice answers (Yes/No/Not Applicable) with short free text questions to allow respondents to elaborate where appropriate.

The questionnaire was developed offline in Word and then formatted in *Google Forms.*^5^ We also provided in Google Forms a brief introduction providing an overview of the ERBFacility COST Action and practical guidance on filling in and submitting the questionnaire.

Using *Google Forms* settings, we added a box for respondents to consent to use of personal data (in compliance with the EU General Data Protection Regulation, GDPR), set up automated email confirmation of successful submission and added a check-box option for respondents to receive a copy of their questionnaire response by email.

### 3. Issuing the questionnaire and supporting documents

We emailed 178 target collections in 38 countries with a concise cover email containing the link to the online *Google Forms* questionnaire and three attachments: (a) a letter from the ERBFacility Chair stressing the importance of the survey (to encourage response); (b) a concise summary of ERBFacility (to provide necessary context) and (c) a list of the institutions to which the questionnaire was being sent (to instill a sense of community). We set a response deadline allowing respondents approximately 2 weeks to complete the questionnaire.

### 4. Tracking and encouraging responses

We tracked responses using a function in *Google Forms* that allows for download of preview results, identifying respondents and maintaining an up-to-date mailing list of institutions whose replies were pending. We issued a number of reminder emails, thanking those who had completed the questionnaire and encouraging others to respond. We extended the deadline once by 2 weeks to allow for additional responses to be submitted.

Following the amended deadline, we downloaded from *Google Forms* a final Excel file containing the responses. We then analyzed the responses in Excel.

## Results

**Fig. 1:**
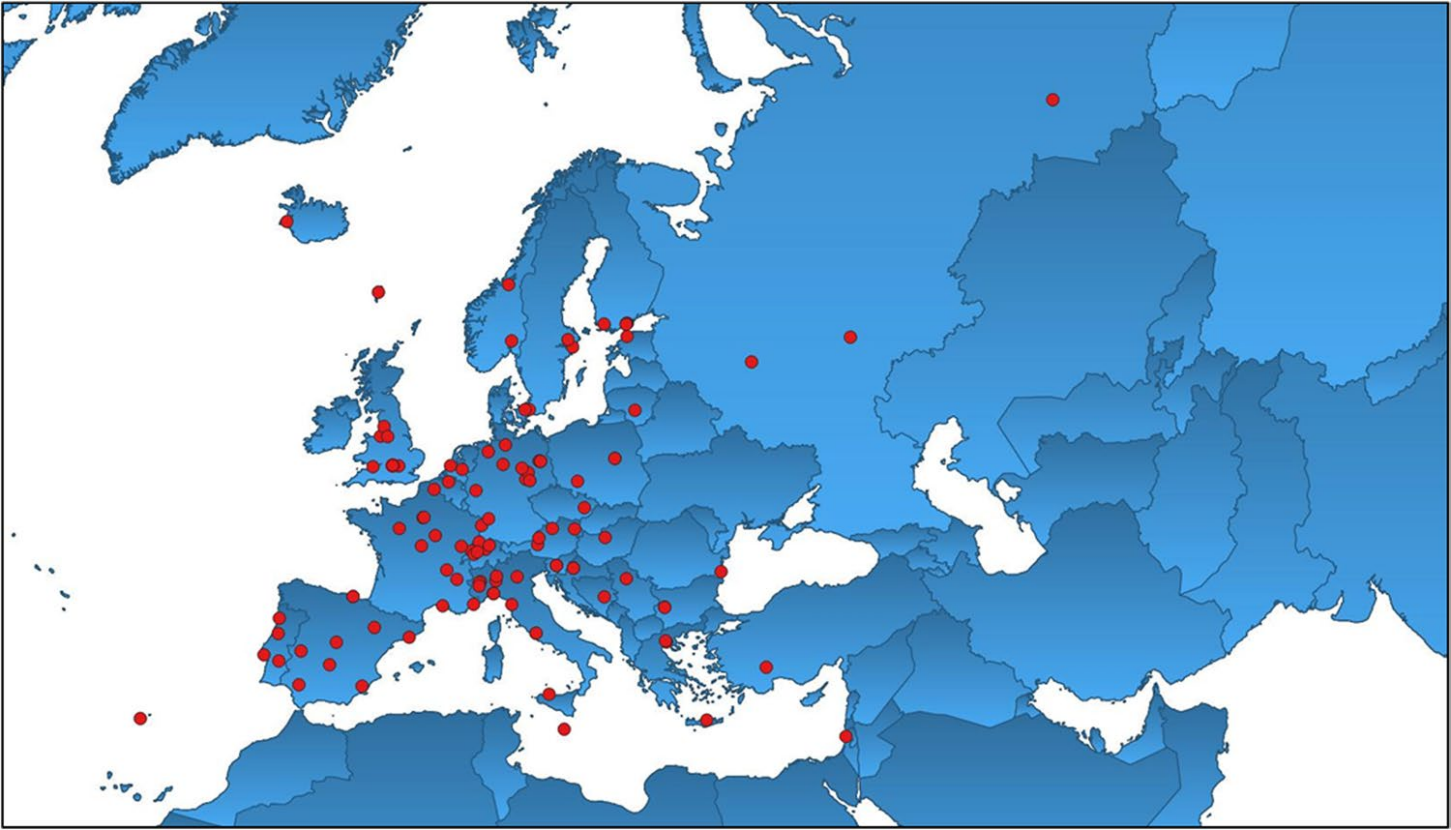
Geographic spread of responding collections (red dots)

We received a total of 116 responses (65% response rate) of which 74 from NHMs, 7 from ESBs and 35 from ORCs (Table 1) from 30 COST countries (plus Russia): Austria, Belgium, Bosnia and Herzegovina, Bulgaria, Croatia, Czech Republic, Denmark, Estonia, Finland, France, Germany, Greece, Hungary, Iceland, Israel, Italy, Lithuania, Malta, Norway, Poland, Portugal, Romania, Russia, Serbia, Slovenia, Spain, Sweden, Switzerland, The Netherlands, Turkey and UK (Fig. 1). For GDPR reasons, contact details for respondents are not given.

**Table 1:** List of the 116 responding institutions

Biologiezentrum des Oberösterreichischen Landesmuseums—Linz	Austria
Haus der Natur—Salzburg	Austria
Naturhistorisches Museum Wien	Austria
Royal Belgian Institute of Natural Sciences—Brussels	Belgium
University of Antwerp, BECO group	Belgium
National Museum of Bosnia and Herzegovina—Sarajevo	Bosnia and Herzegovina
Bulgarian Society for the Protection of Birds—Sofia	Bulgaria
National Museum of Natural History (BAS)—Sofia	Bulgaria
Croatian Natural History Museum—Zagreb	Croatia
Institute of Ornithology, Croatian Academy of Sciences and Arts—Zagreb	Croatia
Institute of Ornithology, Croatian Academy of Sciences and Arts—Zagreb	Croatia
Museum Komenského Přerov (Ornitologická stanice)	Czech Republic
Aarhus University	Denmark
Faroe Islands Museum of Natural History—Hoyvík	Denmark
Natural History Museum of Denmark—Copenhagen	Denmark
Estaonian University of Life Sciences—Tartu	Estonia
Estonian Museum of Natural History—Tallinn	Estonia
Ecosystems and Environment Research Programme—Helsinki	Finland
Finnish Museum of Natural History, University of Helsinki	Finland
Natural Resources Institute Finland—Helsinki	Finland
University of Turku (Department of Biology)	Finland
Ifremer	France
Institut de recherches de la Tour du Valat—Arles	France
Musée des Confluences—Lyon	France
Musée d’histoire naturelle de Lille	France
Musée Vert – Muséum d’histoire naturelle du Mans	France
Musée zoologique de Strasbourg	France
Muséum d’Auxerre	France
Muséum de Bourges	France
Muséum d’histoire naturelle de Grenoble	France
Muséum d’histoire naturelle de Nice	France
Muséum National d’Histoire Naturelle (MNHN)—Paris	France
University of Franche-Comté—Bresançon	France
Fraunhofer IME—Aachen	Germany
German Environment Agency—Dessau	Germany
Landesmuseum Natur und Mensch Oldenburg (State Museum Nature and Man)	Germany
Leibniz Institut for Zoo and Wildlife Research	Germany
MEROS—Halle	Germany
Museum für Naturkunde Berlin	Germany
Museum für Naturkunde Magdeburg	Germany
Naturkundemuseum Stadt Leipzig	Germany
Niedersächsisches Landesmuseum Hannnover	Germany
Staatliches Museum für Naturkunde Karlsruhe	Germany
Universität Hamburg, CeNak, Zoologisches Museum	Germany
Zoological Research Museum Alexander Koenig (ZFMK)—Bonn	Germany
Aristotle University of Thessaloniki	Greece
Natural History Museum of Crete (University of Crete)—Iraklio	Greece
Veterinary Research Institute/Hellenic Agricultural Organisation Demeter—Thermi	Greece
Hungarian Natural History Museum—Budapest	Hungary
Icelandic Institute of Natural History—Garðabær	Iceland
University of Iceland—Reykjavík	Iceland
The Steinhardt Museum of Natural History – Tel Aviv	Israel
Antarctic Environmental Specimen Bank—Genoa	Italy
Dipartimento di Scienze della Terra (University of Torino)	Italy
Istituto Superiore per la Protezione e la Ricerca Ambientale (ISPRA) – Ozzano dell’Emilia	Italy
Italian National Antarctic Museum (MNA, Section of Genoa)	Italy
Museo Civico di Storia Naturale “G. Doria”—Genoa	Italy
Museo Civico di Storia Naturale di Carmagnola (TO)	Italy
Museo Civico di Storia Naturale di Verona	Italy
Museo Civico di Zoologia (MCZR)—Roma	Italy
Museo di Storia Naturale dell’Università di Pavia (MSNPV)	Italy
Museo di Storia Naturale di Milano	Italy
Museo Regionale Scienze Naturali di Torino	Italy
Museum of Zoology P. Donderlein of UNIPA—Palermo	Italy
Natural History Museum (University of Pisa)	Italy
Ornis italica—Roma	Italy
Università degli Studi di Genova—Scuola di Scienze Matematiche, Fisiche e Naturali	Italy
Kaunas Zoological Museum	Lithuania
National Museum of Natural History (Heritage Malta)—L-Imidina	Malta
Natural History Museum (University of Oslo)	Norway
Norwegian University of Science and Technology (NTNU)—Trondheim	Norway
Museum and Institute of Zoology (MZPW for c) – Warsaw	Poland
Museum of Natural History (Wrocław University)	Poland
Society Falcon—Warsaw	Poland
Museu da Ciência da Universidade de Coimbra	Portugal
Museu de História Natural do Funchal	Portugal
Museu de História Natural e das Ciências da Universidade do Porto	Portugal
Museu Nacional de História Natural e da Ciência (Universidad de Lisboa)	Portugal
University of Evora	Portugal
Alexandru Ioan Cuzza (University of Iasi)	Romania
Sibeco Center & Koltsov Institute of Developmental Biology of Russian Academy of Sciences—Berdsk	Russia
State Darwin Museum—Moscow	Russia
Ulyanovsk Regional Museum of Local Lore named after I.A.Goncharov	Russia
Natural History Museum—Belgrade	Serbia
Institute of poultry, birds, small mammals and reptiles—Ljubljana	Slovenia
Slovenian Museum of Natural History—Ljubljana	Slovenia
Biscay Bay Environmental e Biospecimen Bank – Plentzia-Bizkaia	Spain
Estacion Biológica de Doñana—Sevilla	Spain
Institute for Game and Wildlife Research—Ciudad Real	Spain
Institute of Environmental Assessment and Water Research—Barcelona	Spain
National Museum of Natural Sciences (MNCN), Spanish National Research Council (CSIC)—Madrid	Spain
Natural Sciences Museum of Barcelona	Spain
Plentzia Marine Station PiE-UPV/EHU	Spain
Pyrenean Institute of Ecology—Zaragoza	Spain
Universidad de Extremadura (Facultad de Veterinaria)	Spain
University of Murcia	Spain
Department of Environmental Research & Monitoring—Stockholm	Sweden
Museum of Evolution (Uppsala University)	Sweden
Swedish Museum of Natural History—Department of Zoology (Birds)—Stockholm	Sweden
Muséum d’histoire naturelle de Neuchâtel	Switzerland
Natural History Museum Fribourg	Switzerland
Naturhistorisches Museum Basel	Switzerland
Naturhistorisches Museum Bern	Switzerland
Swiss Ornithological Institute – Schweizerische Vogelwarte Sempach	Switzerland
Vulture Conservation Foundation—Zurich	Switzerland
Naturalis Biodiversity Center—Leiden	The Netherlands
Wageningen University, depot Toxicology	The Netherlands
Mehmet Akif Ersoy University, Laf of Ornithology—Burdur	Turkey
Centre for Ecology & Hydrology—Bailrigg	UK
Environmental Research Institute—Thurso	UK
Manchester Museum (University of Manchester)	UK
Amgueddfa Cymru, National Museum Wales—Cardiff	UK
National Museums Liverpool	UK
National Museums Scotland	UK
Oxford University Museum of Natural History	UK
The Natural History Museum, London	UK

Responses were analyzed under each of the five topics (see “Materials and method”). As not all respondents answered every question, the total number of responses (*n*) for each question varies. Responses were disaggregated by type of collection (NHM, ESB, ORC). We provide an overview in this paper of responses to parts B, C, D and E of the survey.

### Receipt/collection of fresh (contemporary) specimens and storage of samples from these specimens

Among NHMs (*n* = 74), 66 (89%) receive fresh raptor specimens, among ESBs (*n* = 7) only 2 (29%) receive fresh raptor specimens, while among ORCs (*n* = 35), 19 (54%) receive fresh raptor specimens.

For NHMs (*n* = 59), the most frequently cited objective for gathering fresh raptor specimens is ‘scientific collection’ (34 = 58%), followed by ‘research’ (22 = 37%) and ‘exhibition’ (13 = 22%); whereas for ESBs (*n* = 1), ‘research’ is the most frequently cited objective and for ORCs (*n* = 23), ‘research’ is the most frequently cited objective (16 = 70%).

Among NHMs (*n* = 68), the receipt of fresh raptor specimens is purely responsive (i.e. specimens are received ad hoc) for 40 (59%), both responsive and planned (i.e. following a collecting strategy) for 26 (38%), and purely planned for 2 (3%). Among ORCs (*n* = 24), receipt is responsive and planned for 14 (58%), responsive only for 6 (25%) and planned only for 4 (17%). For the one responding ESB, receipt is responsive.

Among NHMs (*n* = 63), 52 (82%) receive fresh raptor specimens both from the public and from professionals, 8 (13%) only from professionals and 3 (5%) only from the public. Among ORCs (*n* = 23), 11 (48%) receive specimens from professionals only, 10 (43%) from both the public and professionals and 2 (9%) from the public only. ESBs (*n* = 2) receive specimens from professionals or from both the public and professionals. These raptor specimens include victims of collision (vehicle, window, windmill, power line, etc.), birds found shot, poisoned or otherwise, and ill or injured birds that have died at recovery centres.

Among NHMs (*n* = 64), 5 (8%) receive no specimens, 13 (20%) receive < 10 raptor specimens per annum, 41 (64%) receive 10–99 specimens per annum, 4 (6%) receive 100–1000 per annum and one receives > 1000 per annum. These 64 NMHs receive between them a total of c. 4000 specimens per annum. Among ORCs (*n* = 26), 5(19%) receive < 10 specimens per annum, 19 (73%) receive 10–100 specimens per annum and 2 (8%) receive 100–1000 specimens per annum. These 26 ORCs receive between them a total of c. 1400 specimens per annum. The one responding ESB receives on average 255 specimens per annum. The 91 responding collections (NHMs, ORCs and ESBs) between them thus receive a total of c. 5650 specimens per annum.

Among NHMs (*n* = 66), 39 (59%) receive fresh specimens from the whole country, while 27 (41%) receive specimens from selected regions only. For ORCs (*n* = 25), 14 (56%) receive specimens from certain regions only, while 11 (44%) receive specimens from the whole country. The only ESB responding to this question received specimens from the whole country.

The majority of NHMs and ORCs transport carcasses from the field at ambient temperature, and then store them at minus 20 °C or lower, which is appropriate for storage of tissue destined for contaminant analyses. The one responding ESB both transfers and stores at minus 20 °C or lower.

Among NHMs (*n* = 72), 57 (79%) keep an electronic or paper record of carcasses in their freezers. Among ORCs (*n* = 23), 14 (61%) keep an electronic or paper record. The one responding ESB keeps an electronic or paper record.

Among NHMs (*n* = 56), 7 (13%) had zero raptor carcasses currently in their freezers, 22 (39%) had 1–10 specimens, 29 (52%) had 11–100 specimens, 3 (5%) had 101–250 specimens, 3 (5%) had 251–500 specimens, 2 (4%) had 501–1000 specimens and one had > 1000 specimens (actually > 2600). Assuming a mean value for each range, in total, these 56 responding NHMs stored c. 6650 raptor specimens in their freezers at time of reporting. This number of 6650 specimens in 56 NHMs (arising from several years of collection) is not inconsistent with the estimate above of 4000 specimens arriving at 64 NHMs annually, taking into account that many of the carcasses arriving at NHMs are discarded (see below).

Among ORCs (*n* = 13), 3 (23%) had zero specimens in their freezers, 4 (31%) had 1–10 specimens, 2 (15%) had 11–100 specimens, 1 (8%) had 101–250 specimens, 2 (15%) had 251–500 specimens, none had 501–1000 specimens and 1 (8%) had > 1000 specimens (actually 1640). Assuming a mean value for each range, in total these 13 responding ORCs stored c. 2750 raptor specimens in their freezers at time of reporting. This number of 2750 specimens in 13 ESBs (arising from several years of collection) is not inconsistent with the estimate above of 1400 specimens arriving at 26 ORCs annually, taking into account that many of the carcasses arriving at ORCs are discarded (see below).

The one ESB responding on number of specimens in their freezers had 300 specimens (150 raptors, 150 owls).

Thus, assuming a mean value for each range, all collections together (NHMs, ORCs and ESB, *n* = 70) stored c. 9700 raptor and owl specimens (at time of responding, December 2018–January 2019).

A total of 35 NHMs provided further detail on the breakdown of specimens per species in their freezers. Carcasses of 49 different species are stored across these 35 NHMs, almost all of which are native European species. The species most frequently stored (each > 100 specimens) are Eurasian sparrowhawk *Accipiter nisus*, Eurasian kestrel *Falco tinnunculus*, Eurasian buzzard *Buteo buteo*, tawny owl *Strix aluco*, long-eared owl *Asio otus*, Ural owl *Strix uralensis*, barn owl *Tyto alba* and eagle owl *Bubo bubo* (Tables 2 and 3).

**Table 2:** Total number of individuals (*n*) of diurnal raptors in the freezers of the 35 NHMs

English name	Scientific name	*n*	% of total specimens in the 35 NHMs
Eurasian sparrowhawk	*Accipiter nisus*	134	18.9
Kestrel	*Falco tinnunculus*	107	15.1
Eurasian buzzard	*Buteo buteo*	101	14.2
Northern goshawk	*Accipiter gentilis*	96	13.5
Lesser kestrel	*Falco naumanni*	51	7.2
White-tailed sea-eagle	*Haliaeetus albicilla*	37	5.2
Osprey	*Pandion haliaetus*	19	2.7
Peregrine falcon	*Falco peregrinus*	19	2.7
European honey buzzard	*Pernis apivorous*	18	2.5
Golden eagle	*Aquila chrysaetos*	16	2.2
Cinereous vulture	*Aegypius monachus*	13	1.8
Short-toed snake-eagle	*Circaetus gallicus*	13	1.8
Black kite	*Milvus migrans*	12	1.7
Eurasian hobby	*Falco subbuteo*	7	1.0
Rough-legged buzzard	*Buteo lagopus*	6	0.8
Merlin	*Falco columbarius*	6	0.8
Western marsh-harrier	*Circus aeruginosus*	6	0.8
Hen harrier	*Circus cyaneus*	6	0.8
Booted eagle	*Hieraaetus pennatus*	6	0.8
Eastern imperial eagle	*Aquila heliaca*	5	0.7
Saker falcon	*Falco cherrug*	5	0.7
Montagu’s harrier	*Circus pygargus*	5	0.7
Red kite	*Milvus milvus*	3	0.4
Collared falconet	*Microhierax caerulescens*	3	0.4
Griffon vulture	*Gyps fulvus*	2	0.3
Lesser spotted eagle	*Clanga pomarina*	2	0.3
Bonelli’s eagle	*Aquila fasciata*	2	0.3
Lanner falcon	*Falco biarmicus*	2	0.3
Red-tailed hawk	*Buteo jamaicensis*	1	0.1
Bearded vulture	*Gypaetus barbatus*	1	0.1
Palm-nut vulture	*Gypohierax angolensis*	1	0.1
Greater spotted eagle	*Clanga clanga*	1	0.1
Steller’s sea-eagle	*Haliaeetus pelagicus*	1	0.1
Black-winged kite	*Elanus caeruleus*	1	0.1
Rüppell’s vulture	*Gyps rueppelli*	1	0.1
	**Total number of specimens**	**709**	**100**
	**Total number of species**	**35**

**Table 3:** Total number of individuals (*n*) of owls in the freezers of the 35 NHMs

Common name	Scientific name	*n*	% of total specimens in the 35 NHMs
Tawny owl	*Strix aluco*	167	18.1
Long-eared owl	*Asio otus*	149	16.1
Ural owl	*Strix uralensis*	146	15.8
Barn owl	*Tyto alba*	108	11.7
Eagle owl	*Bubo bubo*	106	11.5
Boreal owl	*Aegolius funereus*	63	6.8
Little owl	*Athene noctua*	54	5.8
Eurasian pygmy owl	*Glaucidium passerinum*	53	5.7
Short-eared owl	*Asio flammeus*	24	2.6
Scops owl	*Otus scops*	17	1.8
Great grey owl	*Strix nebulosa*	17	1.8
Hawk owl	*Surnia ulula*	15	1.6
Snowy owl	*Bubo scandiacus*	4	0.4
Rock eagle owl	*Bubo bengalensis*	1	0.1
	**Total number of specimens**	**924**	**100**
	**Total number of species**	**14**	

Among NHMs (*n* = 43), 21 (49%) permanently store both fresh and dry tissues deriving from raptor carcasses, 16 (37%) store only fresh tissues and 6 (14%) store only dry tissues. Among ORCs (*n* = 18), 14 (78%) store both fresh and dry tissues, 2 (11%) store only fresh tissues and 2 (11%) store only dry tissues. The one responding ESB stores both fresh and dry tissues. The most frequently mentioned tissue samples are the following: muscle, bones, liver, feathers and eggs.

Among NHMs (*n* = 65), 34 (52%) record biometric data. Among responding ORCs (*n* = 23), 8 (35%) record biometric data. The one responding ESB records biometric data. Biometric data typically include wing length, weight, beak length and tarsus length and can be useful supplementary information for contaminant studies — for example providing a guide to the nutritional status of the bird which might affect residue levels. The use of harmonized protocols across collections for biometrics is important but we did not gather any data on this.

Among NHMs (*n* = 37), 21 (57%) retain fresh tissues only in ethanol, 12 (32%) in both ethanol and frozen and 4 (11%) only frozen. Among ORCs (*n* = 18), all but one store tissues frozen. The one responding ESB stores tissues frozen. Tissues retained in ethanol will be of less value for contaminant analysis.

Among NHMs (*n* = 42), 23 (55%) follow protocols when preparing tissues for storage, 18 (43%) do not. Among ORCs (*n* = 20), 13 (65%) follow particular protocols to prepare tissues for storage and 7 (35%) do not. The two responding ESBs follow protocols. Our data does not provide information on the nature of the protocols used, or the extent to which these are harmonized between collections. Standard protocols for preparation and storage of tissues destined for contaminant analyses (e.g. Espín et al. 2020) are important to avoid secondary contamination.

Among NHMs (*n* = 33), 19 (58%) destroy specimens that are not stored and do not donate them to other institutions, 9 (27%) both donate and destroy specimens and 5 (15%) donate and do not destroy. Among ORCs (*n* = 16), 8 (50%) destroy specimens and do not donate, 7 (44%) donate and do not destroy and 1 (6%) both donates and destroys. The one responding ESB donates the specimens it does not store.

Among NHMs (*n* = 66), 58% provide raptor tissue samples to other NHMs, ESBs or ORCs for analysis or storage, 42% do not. Among ORCs (*n* = 23), 35% provide raptor tissue samples to other NHMs, ESBs or ORCs for analysis or storage, 65% do not. The one responding ESB gives samples to various institutions, particularly universities and laboratories. These local networks may be of value for any pan-European networking activity.

### Constraints to receiving/collecting, processing and storage of fresh specimens

Freezer capacity is a constraint to the short-term storage of raptor carcasses for 31 (45%) of NHMs (*n* = 69) and for 11 (50%) of ORCs (*n* = 22). The one responding ESB is not constrained by freezer capacity.

Among NHMs (*n* = 65), long-term storage of fresh tissues is constrained by freezer capacity for 10 (15%) and is not so constrained for 31 (48%) (37% answered ‘not applicable’ –– these were probably collections that do not store frozen tissues long term). Among ORCs, (*n* = 22) long-term storage of fresh tissues is constrained by freezer capacity for just 4 (18%). The two responding ESBs are not constrained by freezer capacity. Long-term storage of fresh tissues is constrained by processing effort for 36% of NHMs (*n* = 64) and for 32% of ORCs (*n* = 22). The two responding ESBs are not constrained by processing effort. The relative lack of constraint here, compared with freezer capacity constraint for whole carcasses, is probably due to the relatively small size of tissue samples, compared to whole carcasses.

Among NHMs (*n* = 69), 35 (51%) have constraints other than freezer capacity and processing effort to the reception and storage of fresh specimens. Among ORCs (*n* = 22), 6 (27%) have other constraints. The two responding ESBs do not have other constraints. Other than processing effort (i.e. lack of staff and/or staff time), the most frequently cited constraint is legislation. A separate paper on constraints related to the sampling of raptors has recently been submitted for publication (Dulsat-Masvidal et al. 2021).

### Historical archives

For questions on the historical archives, some respondents provided separate figures for diurnal raptors and owls, and some provided a single combined figure for diurnal raptors plus owls.

For NHMs providing figures for diurnal raptors only (*n* = 10), the mean number of tissue samples stored is 327. For NHMs providing figures for owls only (*n* = 10), the mean number of tissue samples stored is 235. For NHMs providing combined figures for both diurnal raptors and owls (*n* = 40), the mean number of tissue samples stored is 785.

For ORCs providing figures for diurnal raptors only (*n* = 2), the mean number of tissue samples stored is 6033. For ORCs providing figures for owls only (*n* = 2), the mean number of tissue samples stored is 2477. For ORCs providing combined figures for both diurnal raptors and owls (*n* = 20), the mean number of tissue samples stored could not be calculated due to imprecise answers, but is probably in the low thousands. The one responding ESB stores 13,373 raptor tissue samples and 6372 owl tissue samples.

Among NHMs (*n* = 74), 14 (19%) have digitized their collection and made it available online (i.e. externally), 16 (22%) have digitized but not yet made available online, 32 (43%) have digitization in progress and 12 (16%) have not begun to digitize. Among ORCs (*n* = 23), 1 (4%) has digitized its collection but not yet made it available online, 4 (18%) are digitizing their collections and 18 (78%) have not yet begun to digitize. Of the two ESB respondents, one has digitized its collection and made it available online, and the other has digitized but not yet made it available online.

Among NHMs (*n* = 31), 7 (22%) began storing tissues (other than skins) before 1990, 12 (39%) after 1990 and 12 (39%) after 2000. Among ORCs (*n* = 5), 2 (40%) began storing tissues before 1990 and 3 (60%) after 1990. The one responding ESB started storing tissue samples in 1965.

### Related studies

Among NHMs (*n* = 74), 20 (27%) are actively involved in research on raptors, with 48 (65%) not actively involved (8% ‘not applicable’). Among ORCs (*n* = 31), 26 (84%) do research on raptors, with 4 (13%) not actively involved (3% ‘not applicable). Among ESBs (*n* = 4), 1 (25%) does research on raptors, with 2 (50%) not actively involved (25% ‘not applicable’).

Among NHMs (*n* = 30), 24 (80%) set certain requirements for third parties to access samples (e.g. formal letter or e-mail, formal request to the museum director, submission of a project) and 6 (20%) don’t set any requirements. The one responding ORC does not set any requirements. ESBs did not respond on this issue.

Among NHMs (*n* = 16), 11 (69%) focus their research on diurnal raptors and owls, 4 (25%) only on diurnal raptors and 1 (6%) only on owls. For these NHMs, of the Accipitriformes and Falconiformes, the five most studied species (in descending order) are the following: common buzzard, peregrine falcon *Falco peregrinus*, Northern goshawk *Accipiter gentilis*, white-tailed eagle *Haliaeetus albicilla* and Eurasian sparrowhawk; and of the Strigiformes, the five most studied species are the following: tawny owl, Eurasian scops-owl *Otus scops*, barn owl, Ural owl and long-eared owl. Among ORCs (*n* = 25), 13 (52%) focus their research on both diurnal raptors and owls, 10 (40%) on diurnal raptors only and 2 (8%) only on owls. For these ORCs, of the Accipitriformes and Falconiformes, the five most studied species (in descending order) are the following: white-tailed eagle, Eurasian kestrel, common buzzard, Eurasian griffon *Gyps fulvus* and cinereous vulture *Aegypius monachus*; and of the Strigiformes: Eurasian eagle-owl, tawny owl, barn owl, little owl *Athene noctua* and Ural owl. The one responding ESB focuses its research on diurnal raptors, particularly white-tailed eagle, osprey *Pandion haliaetus* and golden eagle *Aquila chrysaetos*.

For NHMs (*n* = 27), the fields of raptor research most frequently addressed are ecology, genetics, taxonomy and ecotoxicology; for ORCs (*n* = 64), ecotoxicology, ecology, genetics and behaviour and for ESBs (*n* = 2), ecotoxicology. Among NHMs (*n* = 24), 3 (12%) and among ORCs (*n* = 22), 14 (63%) have published ecotoxicological papers. The one responding ESB has published ecotoxicological papers. Among NHMs (*n* = 16), 9 (56%) gave a contact for ecotoxicological studies. At NHMs (*n* = 6), the most studied substances are metals/semimetals (34%) and PCBs (27%), insecticides (13%); at ORCs (*n* = 16), metals/semimetals (22%), PCBs (16%) and insecticides (15%) and at ESBs (*n* = 2), metals/semimetals (17%), PCBs (17%) and flame retardants (17%). Among ORCs (*n* = 22), 21 (95%) gave a contact for ecotoxicological studies. The one responding ESB provided a contact for ecotoxicological studies.

## Discussion and conclusions

The response rate for the questionnaire varied between countries (Table 4). For some countries, all those institutions contacted responded, while for other countries, as few as 25% of the contacted institutions responded. This may introduce some bias into the results though there is no obvious geographical pattern to the response rate.

**Table 4:** Response rate per country

Country	No. of collections to which questionnaire was sent	No. of collections that responded	% of collections that responded
Austria	3	3	100%
Belgium	3	2	67%
Bosnia and Herzegovina	2	1	50%
Bulgaria	2	2	100%
Croatia	3	3	100%
Czech Republic	3	1	33%
Denmark	5	4	80%
Estonia	2	2	100%
Finland	4	4	100%
France	17	12	71%
Germany	28	12	43%
Greece	5	3	60%
Hungary	3	1	33%
Iceland	4	2	50%
Israel	1	1	100%
Italy	18	15	83%
Lithuania	1	1	100%
Malta	2	1	50%
Norway	4	2	50%
Poland	4	3	75%
Portugal	9	5	56%
Romania	4	1	25%
Russia	5	3	60%
Serbia	2	1	50%
Slovenia	2	2	100%
Spain	13	10	77%
Sweden	4	3	75%
Switzerland	7	6	86%
The Netherlands	3	2	67%
Turkey	1	1	100%
UK	14	7	50%
**Total**	**178**	**116**	**65%**

### Collections in Europe receive thousands of raptor carcasses per annum, with NHMs the key recipients for most countries

This survey of raptor collections in Europe shows that collections are an important repository of raptor samples of potential value to pan-European contaminant monitoring. These raptor samples are housed in three types of collection: natural history museums (NHMs), environmental specimen banks (ESBs) and other research institutions (ORCs).

In total, our survey suggests that at least 5500 raptor carcasses arrive annually at NHMs, ESBs and ORCs across Europe, representing a substantial resource for pan-European contaminant monitoring. These carcasses are of species that are nationally protected and cannot be proactively collected and therefore represent an invaluable resource for contaminant research in top predators.

### NHMs are the key recipients of raptor carcasses for most countries

For most countries, NHMs are the main recipients and repositories of these carcasses. Almost 90% of NHMs responding to the survey indicated that they receive fresh raptor carcasses. By contrast, just over half of responding ORCs and only 2 responding ESBs (UK Predatory Bird Monitoring Scheme, Swedish ESB) house raptor samples. Consequently, any pan-European programme for contaminant monitoring in raptors would need to work in most countries with NHMs and ORCs as the key repositories and suppliers of raptor samples.

At present, less than half of responding collections collecting raptor specimens do so for research purposes, suggesting that there is work to be done to raise awareness of the contaminant research and monitoring potential of these specimens.

### NHMs and other collections offer a substantial resource of frozen raptor carcasses and tissues from recent years

Contaminant monitoring typically requires fresh or frozen tissue samples. Our survey confirms that almost all responding collections receiving raptor carcasses (including 94% of NHMs) store these in freezers. The significance of this is that NHMs are potential suppliers of frozen raptor samples going back several years in time, greatly enhancing the number of specimens available and allowing for contaminant monitoring for past years. Indeed, responding collections indicated their freezers housed a total of c. 9700 frozen diurnal raptor and owl carcasses. The total number of raptor carcasses in collections’ freezers across Europe (including those who did not respond) is likely to be larger than this.

This is perhaps one of the key findings of our survey — that, beyond the obvious repositories of frozen raptor carcasses at the two ESBs (UK Predatory Bird Monitoring Scheme, Swedish ESB) and at ORCs (whose *raison d’etre* is often contaminant monitoring), there is a significant resource of frozen raptor carcasses housed in Europe’s NHMs.

Moreover, around 60% of collections that freeze raptor carcasses subsequently retain fresh tissues (e.g. liver, muscle) when processing carcasses (e.g. to add skins to archive collections). While these fresh tissues may often be stored in alcohol (a typical practice in NHMs, but not well suited to contaminant analyses), over half (i.e. 30% of all responding collections) store fresh tissues by freezing. While raptor carcasses in freezers will tend to be of most recent years, frozen tissue samples are likely to extend further back in time the number of years for which frozen raptor samples are available at any one institution.

### Collections have good specimen numbers for species that have been prioritized for pan-European contaminant monitoring

Survey results suggest that the species most frequently kept in freezers are Eurasian sparrowhawk, Eurasian kestrel, Eurasian buzzard, tawny owl, long-eared owl, Ural owl, barn owl and eagle owl. Four of these species — Eurasian kestrel, Eurasian buzzard, tawny owl and barn owl — have been identified as well suited to pan-European contaminant monitoring based on their distribution and key ecological traits (Badry et al. 2020).

### Freezer capacity is a key constraint to retention of carcasses

Our survey suggests that freezer capacity is a key constraint to expanding the role of NHMs as repositories of raptor samples for pan-European contaminant monitoring purposes.

This constraint relates particularly to the temporary storage of (relatively large and space-consuming) raptor carcasses arriving at NHMs, which have to compete for freezer space with carcasses of other bird and other vertebrate species. Indeed, most NHMs are obliged to discard raptor carcasses due to lack of freezer storage capacity. This represents a considerable loss of raptor samples of potential value for pan-European contaminant monitoring.

Freezer capacity is less of a constraint for longer-term storage of tissue samples (e.g. liver, muscle), as these require much less space than whole carcasses. Faster processing of carcasses on arrival at NHMs would reduce demand on freezer space, but for many NHMs, this is constrained by the availability of staff resources. An alternative might be to create a system by which valuable carcasses arriving at NHMs with limited freezer space are shipped to other institutions (NHMs, ESBs or ORCs) with greater freezer and/or processing capacity.

Collections may in the future be persuaded to increase freezer capacity if there is clear demand for frozen raptor specimens for contaminant studies in support of EU chemicals regulations.

### Collections are becoming digitized and thus more accessible

Our survey suggests that collections are making progress on the digitization of their (raptor) collections. Eighty percent of responding collections keep records of carcasses entering their freezers and around half of these have digitized records of frozen carcasses and of tissue samples processed from these carcasses. Digitization of records will be essential to create a pan-European meta-database of samples and thereby make available raptor specimens visible for the purposes of selection for pan-European contaminant monitoring, as well as facilitating exchange of specimens between collections.

### Contaminant biomonitoring is novel for most NHMs

Our survey reveals that around half of all responding collections already do raptor research, but only 12% of responding NHMs are involved in ecotoxicological research on raptors. This suggests that contaminant studies are novel for most NHMs. Work will therefore be required to raise awareness of the importance of such studies and to build capacities in NHMs to engage in such studies. Scientific networks such as ERBFacility and scientific infrastructures such as DiSSCo (Distributed System of Scientific Collections)^6^ can play an important role in promoting and facilitating biomonitoring of contaminants. For example, work is ongoing under ERBFacility to develop a database of European raptor samples, consistent with DiSSCo, to enhance knowledge of and access to available samples.

### Opportunities to optimize raptor collections for pan-European contaminant monitoring

While NHMs offer a large resource of samples to supplement those of ESBs and ORCs for pan-European contaminant monitoring, there is work to be done to optimize the quality of samples for this purpose. Standards and protocols used for sample processing and freezing in NHMs (institutions which are not typically focused on contaminant monitoring) may differ from those used by ESBs and ORCs (institutions focussed on contaminant monitoring). NHM raptor samples may consequently be less suited to monitoring of certain contaminants. For example, cross-contamination during processing may limit the suitability of these samples to monitor contaminants of emerging concern (e.g. personal care products). This caveat might however be addressed by introducing appropriate standards and protocols in NHMs (e.g. building on Espín et al. 2020).

Pan-European contaminant monitoring might most usefully focus on a small number of species that are particularly well suited to this purpose (Badry et al. 2020). However, other species held in collections may also be of value for pan-European contaminant monitoring, for example to study contaminants in specialist food chains. For instance, vultures have been seen to be one of the species most affected by lead (Monclús et al. 2020). Existing raptor collections are likely to be affected by sampling bias and differences in such bias between institutions, and this must be considered in seeking to optimize collections for pan-European contaminant monitoring. Beyond prioritizing certain species, Pan-European monitoring may set other requirements, such as that the samples derive from non-migrant, breeding birds, and/or of specific sex and/or age class. However, once these priorities are known and communicated, it should be possible to motivate collections to prioritize the gathering and storage of specimens of the required species meeting the required parameters. NHMs, ESBs and ORCs thus hold potential to play a key role in the provision of samples for pan-European contaminant monitoring to inform better chemicals management, thereby contributing to the human and environmental health objectives of chemicals regulation.

Building on the findings of this survey, ERBFacility is taking forward a pan-European proof of concept study, which will analyze raptor samples sourced from collections across Europe to reveal spatial patterns for selected contaminants. Our findings also underpin the sourcing of raptor samples across Europe for the LIFE APEX project,^7^ which is working on a number of ‘demonstrators’ on regulatory applications at EU level of contaminant monitoring data from apex predators including raptors. These applications include early warning of emerging contaminants, prioritization of substances for PBT assessment, and assessment of effectiveness of chemical risk management measures.

## Data Availability

The datasets used and/or analyzed during the current study are available from the corresponding author on reasonable request.

## Footnotes

1 NORMAN database system: https://www.norman-network.com/apex

2 IPCHEM: https://ipchem.jrc.ec.europa.eu/RDSIdiscovery/ipchem/index.html

3 www.erbfacility.eu

4 eBEAC: http://www.nhm.ac.uk/research-curation/scientific-resources/collections/zoological-collections/ebeac/index.html

5 https://docs.google.com/forms

6 www.dissco.eu

7 https://lifeapex.eu

## References

[CR1] Badry A, Krone O, Jaspers VLB, Mateo R, García-Fernández A, Leivits M, Shore RF (2020). Towards harmonisation of chemical monitoring using avian apex predators: Identification of key species for pan-European biomonitoring. Sci Total Environ.

[CR2] Dulsat-Masvidal M, Lourenço R, Lacorte S, D’Amico M, Albayrak T, Andevski J, Aradis A, Baltag E, Berger-Tal O, Berny P, Choresh Y, Duke G, Espín S, García-Fernández A, Gómez-Ramírez P, Hallgrimsson GT, Jaspers V, Johansson U, Kovacs A, Krone O, Leivits M, Martínez-López E, Mateo R, Movalli P, Sánchez-Virosta P, Shore RF, Valkama J, Vrezec A, Xirouchakis A, Walker LA, Wernham C (2021) A review of constraints and solutions for collecting raptor samples and contextual data for a pan-European contaminant monitoring scheme. Sci Total Environ 10.1016/j.scitotenv.2021.148599 34328978

[CR3] Espín S, García-Fernández AJ, Herzke D, Shore RF, van Hattum B, Martínez-López E, Coeurdassier M, Eulaers I (2016). Tracking pan-continental trends in environmental contamination using sentinel raptors—What types of samples should we use?. Ecotoxicology.

[CR4] Espín S, Andevski J, Duke G, Eulaers I, Gómez-Ramírez P, Hallgrimsson GT, Helander B, Herzke D et al (2020) A schematic sampling protocol for contaminant monitoring in raptors. Ambio 1–6. 10.1007/s13280-020-01341-9 10.1007/s13280-020-01341-9 PMC7708607 32399779

[CR5] European Commission (2001). Directive 2001/82/EC of the European Parliament and of the Council of 6 November 2001 on the Community code relating to veterinary medicinal products. Off J L.

[CR6] European Commission (2001). Directive 2001/83/EC of the European Parliament and of the Council of 6 November 2001 on the Community code relating to medicinal products for human use. Off J L.

[CR7] European Commission (2004). Directive 2004/28/EC of the European Parliament and of the Council of 31 March 2004 amending Directive 2001/82/EC on the Community code relating to veterinary medicinal products. Off J L.

[CR8] European Commission (2004). Regulation (EC) no 726/2004 of the European Parliament and of the Council of 31 March 2004 laying down Community procedures for the authorisation and supervision of medicinal products for human and veterinary use and establishing a European Medicines Agency. Off J L.

[CR9] European Commission (2006). Regulation (EC) No 1907/2006 of the European Parliament and of the Council of 18 December 2006 concerning the Registration, Evaluation, Authorisation and Restriction of Chemicals (REACH). Off J L.

[CR10] European Commission (2009). Regulation (EC) No 1107/2009 of the European Parliament and of the Council of 21 October 2009 concerning the placing of plant protection products on the market. Off J L.

[CR11] European Union (2012). Regulation (EU) No 528/2012 of the European Parliament and of the Council of 22 May 2012 concerning the making available on the market and use of biocidal products. Off J L.

[CR12] Gómez-Ramírez P, Shore RF, van den Brink NW, van Hattum B, Bustnes JO, Duke G, Fritsch C, García-Fernández AJ (2014). An overview of existing raptor contaminant monitoring activities in Europe. Environ Int.

[CR13] González-Rubio S, Ballesteros-Gómez A, Asimakopoulos AG, Jaspers VL (2021). A review on contaminants of emerging concern in European raptors (2002–2020). Sci Total Environ.

[CR14] Monclús L, Shore RF, Krone O (2020) Lead contamination in raptors in Europe: a systematic review and meta-analysis. Sci Total Environ 141437. 10.1016/j.scitotenv.2020.141437 10.1016/j.scitotenv.2020.141437 32818895

[CR15] Movalli P, Duke G, Osborn D (2008). Introduction to monitoring for and with raptors. Ambio.

[CR16] Movalli P, Dekker R, Koschorreck J, Treu G (2017). Bringing together raptor collections in Europe for contaminant research and monitoring in relation to chemicals regulations. Environ Sci Pollut Res.

[CR17] Movalli P, Krone O, Osborn D, Pain D (2018). Monitoring contaminants, emerging infectious diseases and environmental change with raptors, and links to human health. Bird Study.

[CR18] Movalli P, Duke G, Dekker R, Vrezec A, Shore RF, García-Fernández A, Wernham C, Krone O (2019). Progress on bringing together raptor collections in Europe for contaminant research and monitoring in relation to chemicals regulation. Environ Sci Pollut Res.

[CR19] Vrezec A, Duke G, Kovács A, Saurola P, Wernham C, Burfield I, Movalli P, Bertoncelj I (2012). Overview of raptor monitoring activities in Europe. Acrocephalus.

